# Recovery of intracranial stenoses in varicella zoster virus vasculitis after long-term treatment with valacyclovir and prednisolone

**DOI:** 10.1186/s42466-022-00180-1

**Published:** 2022-05-16

**Authors:** Markus Kraemer, Daniel Strunk, Jana Becker, Roland Veltkamp, Peter Berlit

**Affiliations:** 1 Department of Neurology, Alfried Krupp Hospital, Essen, Germany; 2 Department of Neurology, Medical Faculty, Heinrich-Heine University Düsseldorf, Düsseldorf, Germany; 3 Department of Brain Sciences, Imperial College London, London, UK; 4 German Society of Neurology, Berlin, Germany

**Keywords:** Valacyclovir, Prednisone, VZV vasculitis, Intracranial stenoses

## Abstract

**Background and purpose:**

Optimal treatment of intracranial stenoses in varicella zoster virus (VZV)-associated vasculitis is unknown. This study aims to evaluate the merits and potential pitfalls of a specific therapeutic strategy, initially proposed by Don Gilden in 2015.

**Methods:**

We describe three patients with intracranial stenoses caused by VZV vasculitis successfully treated by a long-term combination of valacyclovir and prednisolone.

**Results:**

All three patients were young men suffering from stroke. Only one reported a first contact to VZV in adulthood. All three presented stenoses in the intracranial part of the internal carotid artery or the proximal segments of the middle cerebral artery as well as an elevated cell count and positive VZV antibody index in cerebrospinal fluid. They received a combination therapy regimen with prednisone and valacyclovir about a minimum of one year. Intracranial stenoses improved markedly in one and almost resolved completely in the other two patients. Side effects of corticosteroid treatment occurred in two patients.

**Conclusions:**

Long-term combination treatment with prednisone and valacyclovir proved to be effective in three young men suffering from intracranial stenosis due to VZV vasculitis.

## Background

Vasculitis associated with varicella zoster virus (VZV) is a rare cause of stroke. It can, however, be assumed that the condition is often not recognized and misdiagnosed [9]. VZV vasculopathy is a common synonym for VZV vasculitis, because in 33% of patients markers of inflammation are absent in cerebrospinal fluid (CSF) studies [22]. VZV was first described as a cause of stroke in 1896 [24] and has been associated with stroke caused by abnormalities in small and large intracranial arteries, aneurysms with or without subarachnoid hemorrhage, dolichoectasia, dissections, and cerebral venous thrombosis. A differentiation is made between VZV vasculitis of small cerebral arteries, in 37% of cases, and large cerebral arteries, reported to be affected in 13% of patients, respectively. Both large and small cerebral arteries were involved in 50% of patients [22]. An intrathecal synthesis of anti-VZV antibodies in combination with the detection of VZV deoxyribonucleic acid (DNA) in the CSF were described in 30% of the patients only, while anti-VZV IgG antibodies in the CSF alone were found in 93%. While positive polymerase chain reaction (PCR) and the detection of anti-VZV antibodies in the CSF are both highly specific, the detection of anti-VZV Ig antibodies in the CSF is the most reliable test to diagnose VZV vasculitis/vasculopathy. Strokes and lesions at the gray-white matter boundary are the hallmark of small-vessel VZV vasculopathy, while stenoses, especially of the M1 segment of the middle cerebral artery (MCA) or the terminal portion of the internal carotid artery (ICA) are characteristic for large-vessel VZV vasculopathy. These findings can be detected in contrast-enhanced high-resolution MRI [4]. Diagnosis of VZV vasculitis can be challenging because CSF pleocytosis is only found in 67% of patients and the interval between the manifestation of the typical VZV rash and stroke can be long [21]. Demographic data, showing an increased risk of stroke after chickenpox in adults and after shingles point to the significance of VZV in the etiology of stroke [15, 17]. Studies also showed an increased risk of stroke, especially after ophthalmic zoster, within the first 5–12 weeks after the acute infection [17]. VZV vasculopathy does not only occur in Human immunodeficiency virus (HIV)-positive pediatric and adult patients, but also in immunocompetent patients. Whether these supposedly immunocompetent patients have a genetic predisposition for VZV vasculopathies should be discussed in the light of the recently reported association between VZV central nervous system (CNS) vasculitis and deficiency in the cytosolic DNA sensor Ribonucleic acid (RNA) Polymerase III [3]. Pathophysiologically, reactivation of latent VZV in cranial nerve ganglia is assumed with VZV spreading along neuronal axons to infect the outermost layer of the arterial wall adventitia. Other findings include thickened intima with thickened myofibroblasts, disrupted internal elastic lamina, and thickened smooth muscle cells [26]. While early VZV vasculitis is associated with the presence of abundant neutrophils in the adventitia, the inflammatory response is reduced over the course of the disease by vascular remodeling, paralleling the changes observed in other cardiovascular and pulmonary vascular diseases [20].

In VZV vasculopathies, it was shown that VZV infection results in significantly increased levels of interleukin 8 in human vascular adventitial fibroblasts, in human perineurial cells, in human cerebral vascular smooth muscle cell, and in human fetal lung fibroblasts [14]. Interleukin 6 levels were only found increased in human vascular cerebral adventitial fibroblasts, in human perineurial cells, and in human fetal lung fibroblasts. Furthermore, other cytokines, including interleukin 2, interleukin 4, interleukin 15, and interleukin 16, TGF-B, neoxanthin 1, neoxanthin 3, IP-10, and CMCP-1, as well as granulocyte macrophage-colony stimulating factor, were also found upregulated in patients with VZV infections [14].

Proper treatment of VZV vasculitis remains unclear and has not yet been widely discussed [6]. Typically, patients with VZV vasculitis are treated with 10–15 mg/kg IV acyclovir for 14 days. Based on the hypothesis of an inflammatory pathogenesis, treatment with prednisolone usually is administered at a dose of 1 mg/kg body weight on the first 5 days of the 14-day course of acyclovir [21]. Long-term corticosteroid treatment of VZV vasculitis seems to be associated with the risk of VZV reactivation [25]. As VZV vasculitis is explained by widespread arterial infection by VZV, the dilemma arises that vasculitic changes and recently produced pro-inflammatory cytokines are indications for immunotherapy, which, however, is problematic without antiviral protection. Furthermore, under short-term antiviral treatment relapses and insufficient improvements especially of intracranial stenoses have been reported [6, 13, 19]. Therefore, a second course of acyclovir or a long-term oral antiviral treatment for several months have been discussed [2, 21]. The optimal immunotherapy and antiviral therapy for VZV have yet to be established.

## Material and methods

In the following, three cases of VZV vasculitis are described which were treated with a long-term corticosteroid and valacyclovir therapy based on the recommendation of the late Don Gilden. An analysis of the demographic data, symptom presentation, CSF and serum parameters as well as response to treatment and side-effect profiles was performed for three consecutive patients with large-vessel vasculitis. This study aims to evaluate the merits and potential pitfalls of a specific therapeutic strategy, initially proposed by Don Gilden in 2015.

## Results

Case 1: A 19-year-old male patient suffered from an episode with amnestic aphasia for 60 min combined with headaches for a few days. He was admitted to another neurological hospital. His neurological examination was normal. Concerning his medical history, he reported inpatient treatment five months before due to chickenpox and Epstein Barr virus infection with a, from the patient’s point of view, good consequent recovery. However, magnetic resonance imaging (MRI) revealed a left sided stroke. Magnetic resonance angiography (MRA) and transcranial ultrasound studies demonstrated left-sided high-grade stenoses of the proximal parts of middle and anterior cerebral arteries, which was revealed in conventional cerebral angiography without any other hints for vasculitis. At that time, his cerebrospinal fluid (CSF) studies showed inflammation with 100 cells per µl and positive VZV antibody index and a positive VZV PCR. There was no oligoclonal banding. Other serum studies including HIV testing and vasculitis screening were negative. He was treated with acyclovir 800 mg five times per day for 14 days and got antiplatelet therapy with aspirin 100 mg per day.

Two months later, he suffered from right-sided prickling of the arm and heavy headaches. There was a new left-sided stroke in the basal ganglia with 8 cells per µl and a positive VZV IgG antibody index in the CSF. A combination therapy with high dose corticosteroid pulses of 1000 mg methylprednisolone for five days and antiviral therapy with valacyclovir 3 times daily for one week was started (Table 1). Afterwards valacyclovir was given once per day. Oral corticosteroids were tapered over 12 months. The patients experienced no further transient or manifest neurological symptoms anymore but suffered from side effects of corticosteroids with an increase in weight of 20 kg. Four months later, no improvement of stenoses was found in ultrasound and MRA studies. One year after the start of valacyclovir and corticosteroids degrees of stenoses markedly decreased and were no longer hemodynamically relevant. After 2 years of valacyclovir treatment with a stable residual stenosis, antiviral therapy was terminated and the patient was monitored yearly without worsening of stenosis or new symptoms.

**Table 1:** Induction therapy in VZV vasculitis

Week	Therapy
Week 0	5 days methylprednisolone 1000 mg intravenously 7 days valacyclovir 1000 mg 3 times per day
Afterwards	Oral prednisolone see Table 2 valacyclovir 1000 mg 1 time per day for more than 1.5 years

**Fig. 1:**
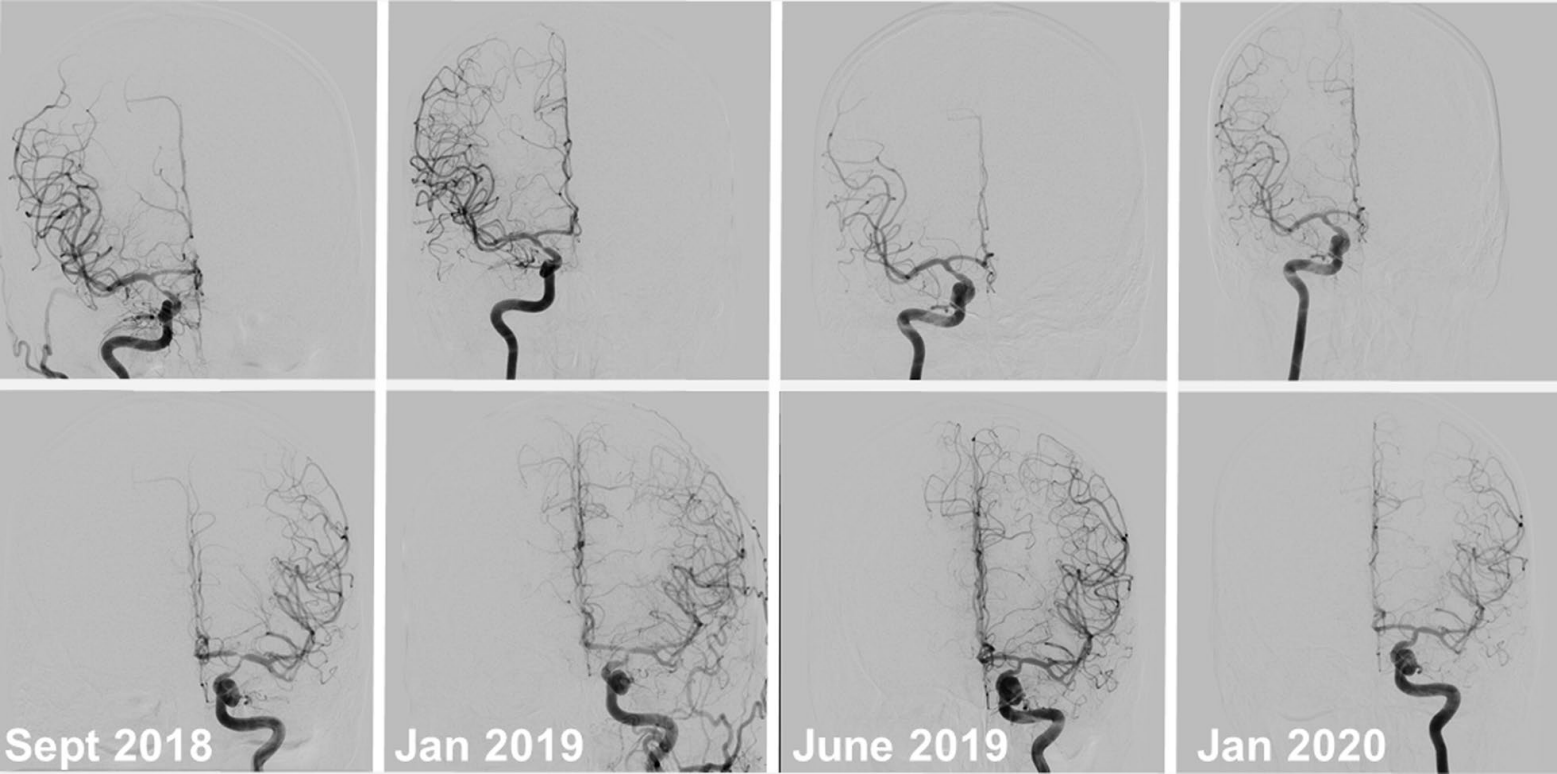
Improvement of bilateral intracranial stenoses in a patient treated with prednisolone and valacylovir

Case 2: A 39-year-old male patient presented with hemiparesis of the right side with hypesthesia. He was diagnosed with ischemic stroke in both MCA territories based on MRI in the regional hospital and got aspirin 100 mg per day. MRA, conventional angiography und duplex ultrasound studies revealed bilateral high-grade stenoses of intracranial ICA and MCA without vasculitic changes of peripheral cerebral arteries and without basal collaterals (Fig. 1). Lumbar puncture showed a pleocytosis of 44 cells per µl. Other examinations including transesophageal echocardiography (TEE), vasculitis screening and HIV testing were completely normal. When the patient was referred to us, the control of CSF studies revealed 28 cells per µl and a very high VZV IgG antibody index of 12.7 (norm < 1.5) as well as specific oligoclonal banding. The patient reported a mild skin rash on both arms four years ago when his daughter had chickenpox infection despite he had chickenpox as a child. We diagnosed VZV vasculitis and gave 1000 mg methylprednisolone for 5 days combined with 1000 mg valacyclovir three times per day for seven days. Afterwards, we tapered the steroids by 10 mg per week starting with 80 mg prednisone and prescribed a permanent treatment with valacyclovir 1000 mg per day. Four months later, angiography demonstrated improvement of the stenoses and CSF studies were improved with a normal cell count, no oligoclonal banding and a decreased VZV antibody index of 3.0. We now tapered oral glucocorticoids by 5 mg every two weeks coming from 30 mg prednisolone down to 10 mg for 4 weeks and 7.5 mg per day for 8 weeks (Table 2). The patient reported no side effects, his weight was stable, and he had no hints for steroid-induced diabetes mellitus or arterial hypertension. On the next follow up 9 months after therapy initiation, further improvements of the stenoses and the VZV antibody index in CSF with 2.0 were detected. We now continued the treatment with valacyclovir 1000 mg per day and tapered down the prednisone by 0.5 mg per month. At last follow up 15 months after treatment initiation, the stenoses of the MCA on both sides and the left-sided intracranial ICA had completely resolved and the right-sided intracranial ICA was almost normal (Fig. 1). The VZV antibody index in CSF was now 1.6 (normal < 1.5). Finally, corticosteroids and valacyclovir were discontinued after 16 months of treatment.

**Table 2:** Potential dosage regimen of oral prednisolone after induction therapy

Dosage prednisolone in mg	Duration in weeks
80	1
70	1
60	1
50	1
40	1
30	2
20	2
15	2
10	4
7.5	8
6	4
5	4
4.5	4
4	4
3.5	4
3	4
2.5	4
2	4
1.5	4
1	4
0.5	4

**Fig. 2:**
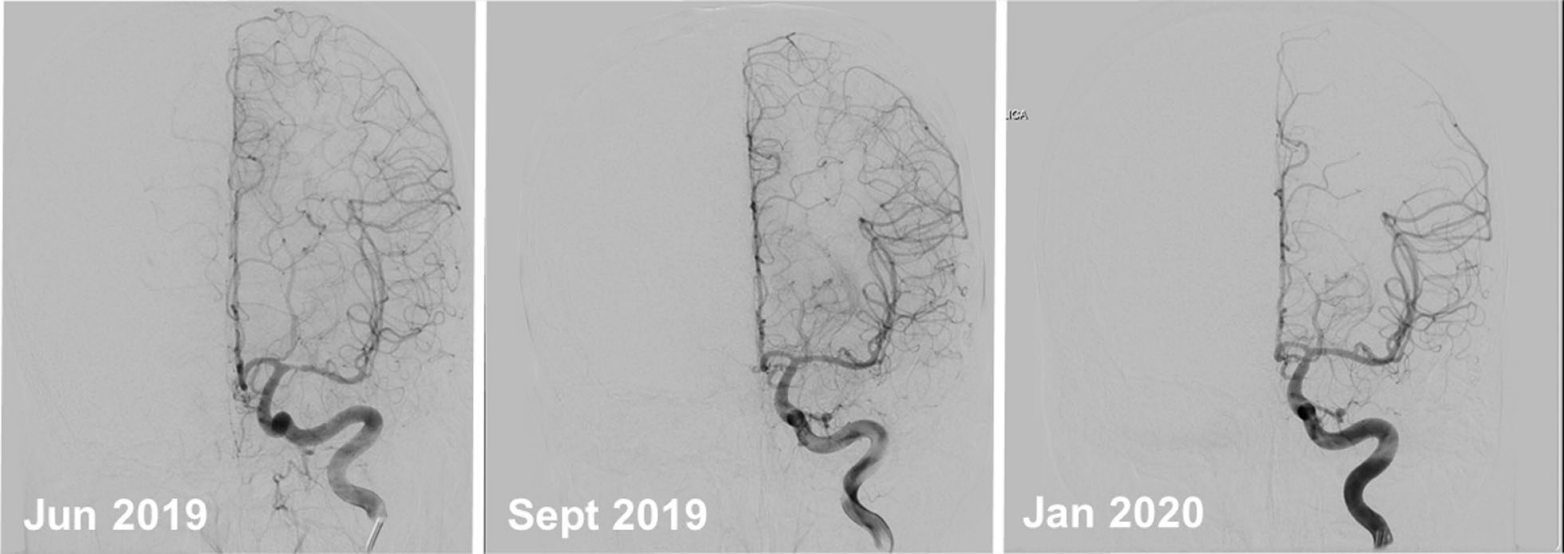
Improvement of left-sided stenosis of middle cerebral artery in a patient treated with prednisolone and valacyclovir

Case 3: A 24-year-old patient was referred to our hospital with suspected CNS vasculitis. Some days earlier, he had suffered a sudden right sided hemiparesis and dysarthria. The patient reported heavy persisting headaches before the onset of stroke, beginning one day after refreshment of anti-rabies inoculation two weeks earlier. Five months earlier, he had suffered from heavy headaches for three days with vomiting with recurrent relapses suggestive of migraine. We diagnosed left-sided MCA stroke in MRI and a high-grade stenosis of the proximal left MCA (M1) in MRA, ultrasound and angiogram studies (Fig. 2). Aspirin 100 mg per day was given. Angiographically, there were no further signs for vasculitis or moyamoya phenomenon. CSF studies depicted inflammation with 33 cells per µl and an elevated VZV antibody index of 5.6. There was identical oligoclonal banding in CSF and in serum. Other examinations were without hints for vasculitis, HIV or other causes for stroke. We diagnosed VZV vasculitis and treated with methylprednisolone 1000 mg per day for five days combined with valacyclovir 1000 mg three times a day for seven days and antiplatelet therapy. Oral corticoid therapy was tapered weekly by 10 mg coming from 80 mg per day. Reaching 30 mg per day, we tapered more slowly reaching 7.5 mg within 10 weeks (Table 2). Valacyclovir 1000 mg per day was continuously given. Three months after the start of the combined treatment, his CSF data were markedly improved with 7 cells per µl and a VZV antibody index of 2.5. Moreover, angiography showed nearly normal cerebral vessels (Fig. 2). However, the patient complained about corticoid side effects with weight increase of 20 kg, steroid-induced acne and stretch marks. Therefore, corticosteroids were reduced faster than originally planned. Four months later, when he took 3 mg prednisone per day, his MCA stenosis had completely decreased, but mild ICA stenosis persisted. CSF studies were still not normal with 8 cells per µl, an elevated VZV antibody index of 3.7 and oligoclonal banding in the CSF. This was why we increased the corticosteroid dose and continued daily valacyclovir. The patient was stable with regard to new strokes and transient ischemic attacks (TIAs) in the further follow up, but experienced episodes interpreted as migraine with aura.

## Discussion

The presented cases show the good response of large-vessel stenoses of proximal intracranial vessels in VZV vasculitis to long-term treatment with corticosteroids and valacyclovir, a regimen recommended by the late Don Gilden. Considering the pro-inflammatory cytokines produced by VZV-infected cerebrovascular cells, this strategy appears reasonable and consistent, though there is no data on the natural course of the disease. Given that VZV vasculitis can cause severe conditions, such as stroke, vessel dissection, aneurysms, and venous sinus thrombosis, early and aggressive treatment seems to be indispensable. Based on the available data, the combination of steroid and antiviral therapy over a prolonged period appears more promising than short-term approaches using this combination as is used to be ten years ago, when corticosteroids were added for only five days [6].

In our opinion, the pleocytosis and pathophysiological background support the combined treatment strategy of prednisolone and valacyclovir proposed by Don Gilden [5–8, 11, 23]. The significant improvement of the intracranial stenoses based on early treatment, as demonstrated in Fig. 1, highlights the importance of early detection and treatment of VZV vasculitis. With the proximal MCA and the terminal ICA as predilection sites, this condition can easily be mistaken as a non-inflammatory disorder, such as moyamoya angiopathy (MMA), especially unilateral MMA, which is—the other way around—often misdiagnosed as vasculitis [1, 12]. However, to identify this differential diagnosis of vasculitis, in particular VZV vasculitis, CSF analysis is necessary in all unclear cerebral vasculopathies [12, 16].

The work-up of unclear intracranial stenoses should include CSF analysis with anti-VZV antibody production [1]. Considering the controversy about the role of VZV in Takayasu arteritis, in GCA and granulomatous aortitis, CSF analysis, including anti-VZV antibodies, could be useful for these conditions as well [8, 10]. It is important to be aware, that the absence of preceding chickenpox or shingles, CSF pleocytosis or VZV Deoxyribonucleic acid (DNA) in CSF does not rule out the diagnosis of VZV vasculopathy.

A limitation of this study is that—which also applies to many other therapies for rare diseases—treatment is based on expert opinion supported by initial data indicating potential efficacy. However, it remains unclear how the natural course of the disease or how effective a lower-dose treatment strategy would have been. Furthermore, the question arises, if there are certain conditions, which need to be fulfilled in order to maximize the likelihood of a favorable outcome, when applying the proposed therapeutic regimen. Clinical experience has underlined the importance of early detection and treatment of VZV vasculitis. This, in turn, leads to the question, how such an early state can be detected reliably in daily routine. In our view, hallmarks of florid inflammation due to VZV vasculitis, a state that can be favorably influenced, are CSF pleocytosis, a marked increase in CSF protein concentration, oligoclonal bands, and a pathological VZV antibody index, hinting at intrathecal synthesis of VZV-IgG-antibodies. On the other hand, according to Don Gilden, a positive CSF VZV PCR is of minor importance. Despite all this, we are well aware of the fact that these findings need to be confirmed in larger cohorts.

Another limitation is that this report does not represent a randomized controlled study. However, in rare diseases, recruitment is difficult as demonstrated in the GACHE study [18]. This multicenter, randomized, double-blind, placebo-controlled trial was intended to analyse adjuvant dexamethason additionally to acyclovir in Herpes-simplex virus encephalitis. It was stopped prematurely for slow recruitment after 41 patients and failed to show significant differences in this small patient group [18].

Moreover, it has to be mentioned that two of our patients experienced significant (but reversible) corticosteroid side effects under long-term therapy. Based on the case series, it cannot be excluded that a shorter corticosteroid treatment or faster tapering would have shown a similar response and improvement of the stenoses. The main limitation of this regimen consists in the commonly known side effects of glucocorticoids, such as weight gain, increased blood glucose, iatrogenic diabetes mellitus, striae rubrae, or other signs of Cushing’s syndrome. Consequently, the intake of oral glucocorticoids, exceeding the threshold of 7.5 mg/d might cause serious health issues for the respective patients. This regimen showed in Table 2 includes a dosage of prednisolone above the Cushing threshold dose of 7.5 mg for 15 weeks. In comparison with this regimen, the prednisone-tapering regimen in giant cell arteritis includes a dosage above 7 mg for 13 weeks in addition to tocilizumab [27], and showed a reduction of side effects compared with former dosage regimens in giant cell arteritis. Thus, a prednisone dosage exceeding 7.5 mg per day in late Gilden’s regimen does not seem to be the main problem. However, the riskiness but perhaps also the effectiveness could consist in the slow tapering below the threshold of a daily dose of 7.5 mg for 13 months, as decrease in weight seemed to be difficult to realize, even under such a lower daily dose of prednisone. As in other intracranial stenoses, antiplatelet therapy is advisable.

On the other hand, the excellent improvement of the intracranial stenoses and, as a result, the decrease in the associated risk of stroke as a result of the administered therapy in our case series are a strong argument speaking in favor of this treatment regimen. Almost all side effects related to corticosteroid medication (despite striae) were reversible, the prolonged valacyclovir therapy appears to be generally well tolerated, and just renal dysfunction should be considered.

## Conclusions

In conclusion and in the absence of a more specific and better compatible treatment, combined long-term corticosteroid and valacyclovir therapy of intracranial stenoses associated with VZV vasculitis/vasculopathy is effective and remarkably safe in a younger population. Appropriate dosage and duration of treatment needs to be determined as well as whether oral treatment is as effective as intravenous application of corticosteroids and antiviral therapy.

## Data Availability

Due to protection of data privacy data cannot be shared.

## Abbreviations

CNS: Central nervous system
CSF: Cerebrospinal fluid
D: Day
DNA: Deoxyribonucleic acid
HIV: Human immunodeficiency virus
ICA: Internal carotid artery
Ig: Immunoglobulin
IV: Intravenous
MCA: Middle cerebral artery
MMA: Moyamoya angiopathy
MRA: Magnetic resonance angiography
MRI: Magnetic resonance imaging
PCR: Polymerase chain reaction
RNA: Ribonucleic acid
TEE: Transesophageal echocardiography
TIA: Transient ischemic attack
VZV: Varicella zoster virus

## References

[1] Berlit P, Kraemer M (2014). Cerebral vasculitis in adults: What are the steps in order to establish the diagnosis? Red flags and pitfalls. Clinical and Experimental Immunology.

[2] Bubak AN, Como CN, Blackmon AM, Jones D, Nagel MA (2018). Varicella zoster virus differentially alters morphology and suppresses proinflammatory cytokines in primary human spinal cord and hippocampal astrocytes. Journal of Neuroinflammation.

[3] Carter-Timofte ME, Hansen AF, Mardahl M, Fribourg S, Rapaport F, Zhang SY, Casanova JL, Paludan SR, Christiansen M, Larsen CS, Mogensen TH (2018). Varicella-zoster virus CNS vasculitis and RNA polymerase III gene mutation in identical twins. Neurol Neuroimmunol Neuroinflamm.

[4] Cheng-Ching E, Jones S, Hui FK, Man S, Gilden D, Bhimraj A, Uchino K (2015). High-resolution MRI vessel wall imaging in varicella zoster virus vasculopathy. Journal of the Neurological Sciences.

[5] Gilden D (2015). Varicella-zoster virus infections. Continuum (Minneap Minn).

[6] Gilden D, Cohrs RJ, Mahalingam R, Nagel MA (2009). Varicella zoster virus vasculopathies: Diverse clinical manifestations, laboratory features, pathogenesis, and treatment. Lancet Neurology.

[7] Gilden D, Grose C, White T, Nagae L, Hendricks RL, Cohrs RJ, Nagel MA (2016). Successful antiviral treatment after 6 years of chronic progressive neurological disease attributed to VZV brain infection. Journal of the Neurological Sciences.

[8] Gilden D, Nagel M, Cohrs R, Mahalingam R, Baird N (2015). Varicella zoster virus in the nervous system. F1000Res.

[9] Gilden D, Nagel MA, Cohrs RJ, Mahalingam R (2013). The variegate neurological manifestations of varicella zoster virus infection. Current Neurology and Neuroscience Reports.

[10] Gilden D, White TM, Nagae L, Gurdin WH, Boyer PJ, Nagel MA (2015). Successful antiviral treatment of giant cell arteritis and takayasu arteritis. JAMA Neurology.

[11] Gilden DH, Bennett JL, Kleinschmidt-DeMasters BK, Song DD, Yee AS, Steiner I (1998). The value of cerebrospinal fluid antiviral antibody in the diagnosis of neurologic disease produced by varicella zoster virus. Journal of the Neurological Sciences.

[12] Graf J, Schwitalla JC, Albrecht P, Veltkamp R, Berlit P, Hartung HP, Aktas O, Kraemer M (2019). Misdiagnoses and delay of diagnoses in Moyamoya angiopathy-a large Caucasian case series. Journal of Neurology.

[13] Haug A, Mahalingam R, Cohrs RJ, Schmid DS, Corboy JR, Gilden D (2010). Recurrent polymorphonuclear pleocytosis with increased red blood cells caused by varicella zoster virus infection of the central nervous system: Case report and review of the literature. Journal of the Neurological Sciences.

[14] Jones D, Neff CP, Palmer BE, Stenmark K, Nagel MA (2017). Varicella zoster virus-infected cerebrovascular cells produce a proinflammatory environment. Neurol Neuroimmunol Neuroinflamm.

[15] Kang JH, Ho JD, Chen YH, Lin HC (2009). Increased risk of stroke after a herpes zoster attack: A population-based follow-up study. Stroke; A Journal of Cerebral Circulation.

[16] Kraemer M, Berlit P (2010). Primary central nervous system vasculitis and moyamoya disease: Similarities and differences. Journal of Neurology.

[17] Lin HC, Chien CW, Ho JD (2010). Herpes zoster ophthalmicus and the risk of stroke: A population-based follow-up study. Neurology.

[18] Meyding-Lamadé U, Jacobi C, Martinez-Torres F, Lenhard T, Kress B, Kieser M, Klose C, Einhäupl K, Bösel J, Mackert MB, Homberg V (2019). The German trial on Aciclovir and Corticosteroids in Herpes-simplex-virus-Encephalitis (GACHE): A multicenter, randomized, double-blind, placebo-controlled trial. Neurological Research and Practice.

[19] Miravet E, Danchaivijitr N, Basu H, Saunders DE, Ganesan V (2007). Clinical and radiological features of childhood cerebral infarction following varicella zoster virus infection. Developmental Medicine and Child Neurology.

[20] Nagel MA, Bennett JL, Khmeleva N, Choe A, Rempel A, Boyer PJ, Gilden D (2013). Multifocal VZV vasculopathy with temporal artery infection mimics giant cell arteritis. Neurology.

[21] Nagel MA, Bubak AN (2018). Varicella zoster virus vasculopathy. The Journal of Infectious Diseases.

[22] Nagel MA, Cohrs RJ, Mahalingam R, Wellish MC, Forghani B, Schiller A, Safdieh JE, Kamenkovich E, Ostrow LW, Levy M, Greenberg B (2008). The varicella zoster virus vasculopathies: Clinical, CSF, imaging, and virologic features. Neurology.

[23] Nagel MA, Forghani B, Mahalingam R, Wellish MC, Cohrs RJ, Russman AN, Katzan I, Lin R, Gardner CJ, Gilden DH (2007). The value of detecting anti-VZV IgG antibody in CSF to diagnose VZV vasculopathy. Neurology.

[24] Nagel MA, Gilden D (2016). Developments in varicella zoster virus vasculopathy. Current Neurology and Neuroscience Reports.

[25] Nagel MA, Jones D, Wyborny A (2017). Varicella zoster virus vasculopathy: The expanding clinical spectrum and pathogenesis. Journal of Neuroimmunology.

[26] Nagel MA, Traktinskiy I, Azarkh Y, Kleinschmidt-DeMasters B, Hedley-Whyte T, Russman A, VanEgmond EM, Stenmark K, Frid M, Mahalingam R, Wellish M (2011). Varicella zoster virus vasculopathy: Analysis of virus-infected arteries. Neurology.

[27] Stone JH, Tuckwell K, Dimonaco S, Klearman M, Aringer M, Blockmans D, Brouwer E, Cid MC, Dasgupta B, Rech J, Salvarani C (2017). Trial of tocilizumab in giant-cell arteritis. The New England Journal of Medicine.

